# Supplementary material for: A widely distributed metalloenzyme class enables gut microbial metabolism of host- and diet-derived catechols
Source: eLife. 2020 Feb 18;9:e50845. doi: 10.7554/eLife.50845 (PMC7028382; doi:10.7554/eLife.50845)
Supplement: Transparent reporting form [file elife-50845-transrepform.docx]

***eLife’s* transparent reporting form**

We encourage authors to provide detailed information *within their submission* to facilitate the interpretation and replication of experiments. Authors can upload supporting documentation to indicate the use of appropriate reporting guidelines for health-related research (see [EQUATOR Network](http://www.equator-network.org/%20)), life science research (see the [BioSharing Information Resource](https://biosharing.org/)), or the [ARRIVE guidelines](http://www.plosbiology.org/article/info:doi/10.1371/journal.pbio.1000412) for reporting work involving animal research. Where applicable, authors should refer to any relevant reporting standards documents in this form.

If you have any questions, please consult our Journal Policies and/or contact us: [editorial@elifesciences.org](mailto:editorial@elifesciences.org).

**Sample-size estimation**

- You should state whether an appropriate sample size was computed when the study was being designed
- You should state the statistical method of sample size computation and any required assumptions
- If no explicit power analysis was used, you should describe how you decided what sample (replicate) size (number) to use

Please outline where this information can be found within the submission (e.g., sections or figure legends), or explain why this information doesn’t apply to your submission:

Information about sample size for experiments can be found in the figure captions. The sample size was at minimum n=3 replicates based on previous similar studies in this field of research; no explicit power analysis was used to determine replicate number

**Replicates**

- You should report how often each experiment was performed
- You should include a definition of biological versus technical replication
- The data obtained should be provided and sufficient information should be provided to indicate the number of independent biological and/or technical replicates
- If you encountered any outliers, you should describe how these were handled
- Criteria for exclusion/inclusion of data should be clearly stated
- High-throughput sequence data should be uploaded before submission, with a private link for reviewers provided (these are available from both GEO and ArrayExpress)

Please outline where this information can be found within the submission (e.g., sections or figure legends), or explain why this information doesn’t apply to your submission:

Information about the replicates and the number of times experiments were performed is present in figure and table captions throughout the main text and supplementary information

Criteria for exclusion are only relevant in Figure 2 and are clearly described in the figure caption

RNA-Seq data has been deposited into the Sequence Read Archive available by way of BioProject PRJNA557713 for *Eggerthella lenta* A2 and PRJNA557714 for *Gordonibacter pamelaeae* 3C.

**Statistical reporting**

- Statistical analysis methods should be described and justified
- Raw data should be presented in figures whenever informative to do so (typically when N per group is less than 10)
- For each experiment, you should identify the statistical tests used, exact values of N, definitions of center, methods of multiple test correction, and dispersion and precision measures (e.g., mean, median, SD, SEM, confidence intervals; and, for the major substantive results, a measure of effect size (e.g., Pearson's r, Cohen's d)
- Report exact p-values wherever possible alongside the summary statistics and 95% confidence intervals. These should be reported for all key questions and not only when the p-value is less than 0.05.

Please outline where this information can be found within the submission (e.g., sections or figure legends), or explain why this information doesn’t apply to your submission:

Description of statistical tests can be found in figure legends throughout the main text and supplemental information, as well as in the source data, which contains details of tests and its parameters

Exact p-values are reported in figure legends and in source data

Individual replicates are shown in graphs in the figures throughout the main text

If replicates are not shown on the graph, the replicate information is provided in the source data file

(For large datasets, or papers with a very large number of statistical tests, you may upload a single table file with tests, Ns, etc., with reference to sections in the manuscript.)

**Group allocation**

- Indicate how samples were allocated into experimental groups (in the case of clinical studies, please specify allocation to treatment method); if randomization was used, please also state if restricted randomization was applied
- Indicate if masking was used during group allocation, data collection and/or data analysis

Please outline where this information can be found within the submission (e.g., sections or figure legends), or explain why this information doesn’t apply to your submission:

No randomization was performed to allocate samples into experimental groups

No masking was used during data collection or analysis

**Additional data files (“source data”)**

- We encourage you to upload relevant additional data files, such as numerical data that are represented as a graph in a figure, or as a summary table
- Where provided, these should be in the most useful format, and they can be uploaded as “Source data” files linked to a main figure or table
- Include model definition files including the full list of parameters used
- Include code used for data analysis (e.g., R, MatLab)
- Avoid stating that data files are “available upon request”

Please indicate the figures or tables for which source data files have been provided:

Source data files for the experiments described in main text figures -1-4 and 7 has been provided. The source data described in these files also include data from the experiments presented in fig. S4, fig. S6, fig. S8, fig. S9, fig. S14, and table S12.

Synthetic compound characterization data (NMR) is present in the supplementary information
